# Analysis of SOX2-Expressing Cell Populations Derived from Human Pluripotent Stem Cells

**DOI:** 10.1016/j.stemcr.2013.09.005

**Published:** 2013-10-31

**Authors:** David A. Brafman, Noel Moya, Stephanie Allen-Soltero, Thomas Fellner, Megan Robinson, Zoë L. McMillen, Terry Gaasterland, Karl Willert

**Affiliations:** 1 Stem Cell Program, Department of Cellular and Molecular Medicine, UCSD, 9500 Gilman Drive, La Jolla, CA 92093-0695, USA; 2 Scripps Genome Center, UCSD and Scripps Institution of Oceanography, 9500 Gilman Drive, La Jolla, CA 92093-0202, USA

## Abstract

SOX2 is involved in several cell and developmental processes, including maintenance of embryonic stem cells, differentiation of neural progenitor cells, and patterning of gut endoderm. To study its role in a human system, we generated a human embryonic stem cell (hESC) line harboring a reporter gene encoding GFP in the *SOX2* locus. This SOX2 reporter line faithfully recapitulates expression of the *SOX2* gene in undifferentiated human pluripotent stem cells (hPSCs), neural progenitor cells (NPCs), and anterior foregut endoderm (AFE). In undifferentiated hESCs, GFP expression corresponds to those cells with highest levels of expression of genes associated with the pluripotent state. In NPCs, expression of GFP can be employed to isolate cells expressing markers associated with NPC multipotency. In AFE, we used transcriptome-wide expression analysis to identify cell surface markers with elevated expression in this population, thereby facilitating isolation and purification of this hPSC-derived cell population.

## Highlights

A SOX2-GFP hESC line is used to isolate and characterize multiple cell types
A cell surface marker signature allows for the purification of endodermal progeny
Targeted gene insertion with adeno-associated virus (AAV) is highly efficient

## Teaser

*SOX2* is expressed during multiple developmental stages. Brafman, Willert, and colleagues used human embryonic stem cells carrying a *SOX2* reporter to characterize its expression in undifferentiated cells and in neural and lung progenitor cells. This approach led to the identification of a cell surface signature to isolate prospective lung progenitors.

## Introduction

Human pluripotent stem cells (hPSCs; including human embryonic stem cells [hESCs] and human induced pluripotent stem cells [hiPSCs]), provide a unique model system to study early human development and generate mature and functional cell types suitable for disease modeling, cell transplantation, and replacement therapies. Clinical applications of hPSCs will require a detailed understanding of the mechanisms that maintain their pluripotency or result in their differentiation to specific lineages. A particularly attractive method to study the underlying mechanisms that control pluripotency and differentiation is through the use of marker cell lines in which specific genes known to function in these processes are modified with a “molecular beacon,” such as a gene encoding a fluorescent protein. Expression of such a tagged gene can be used to analyze and characterize the cells in which expression of this gene is either activated or repressed. Here, we describe the generation and characterization of such a marker line for the gene *SOX2*, which plays multiple roles in hPSC pluripotency and differentiation (Arnold et al., 2011; Lefebvre et al., 2007).

SOX2 is a member of the SRY-related high-mobility-group box (SOX) transcription factors and controls cell fate and differentiation in a variety of cell types during development (Kiefer, 2007; Lefebvre et al., 2007). During the initial stages of development, SOX2 is expressed in the inner cell mass of the blastocyst (Lorthongpanich et al., 2008; Rossant, 2004) and along with OCT4 is responsible for regulating the pluripotent precursors that develop into the three germ layers (Avilion et al., 2003). SOX2 acts in coordination with other factors, such as NANOG (Wang et al., 2006) and OCT4 (Nichols et al., 1998), to maintain ESCs in a pluripotent state. Furthermore, ectopic expression of SOX2 along with OCT4, KLF4, and c-MYC can induce a pluripotent stem cell state from adult human fibroblasts, giving rise to hiPSCs (Takahashi et al., 2007).

In addition to regulating the pluripotent state, SOX2 controls the formation of several cell types during fetal development, such as the nervous system (Ellis et al., 2004), anterior foregut endoderm (Que et al., 2007), and sensory cells of the taste bud and inner ear (Dabdoub et al., 2008; Kiernan et al., 2005; Okubo et al., 2006). SOX2 also regulates the progenitor cells in several adult tissues in the brain (Ellis et al., 2004), trachea (Que et al., 2009), and pituitary glands (Fauquier et al., 2008). A recent genetic lineage tracing study revealed that SOX2 regulates adult stem cells and tissue homeostasis in several adult epithelial tissues in the stomach, cervix, anus, testes, lens, and glands associated with the oral cavity, trachea, and cervix (Arnold et al., 2011).

In this study, we describe the generation and characterization of a hESC line in which the endogenous *SOX2* locus was targeted to express GFP. The targeted reporter line facilitated the flow-cytometry-based purification and genetic assessment of SOX2-positive (SOX2^+^) cells in pluripotent hESCs as well as hESC-derived neural progenitor cells (NPCs) and anterior foregut endoderm (AFE). Genome-wide analysis of SOX2^+^ AFE cells revealed a global gene expression signature that distinguished hESC-derived AFE cells from other cell types. This signature included two cell surface markers that permitted purification of SOX2^+^ AFE cells from differentiating hESC cultures. Therefore, this SOX2-GFP reporter line is a valuable tool to dissect the role of SOX2 in regulating pluripotency, self-renewal, and differentiation.

## Results

### Generation of a SOX2-GFP Reporter hESC Line by AAV Mediated Homologous Recombination

**Figure 1:**
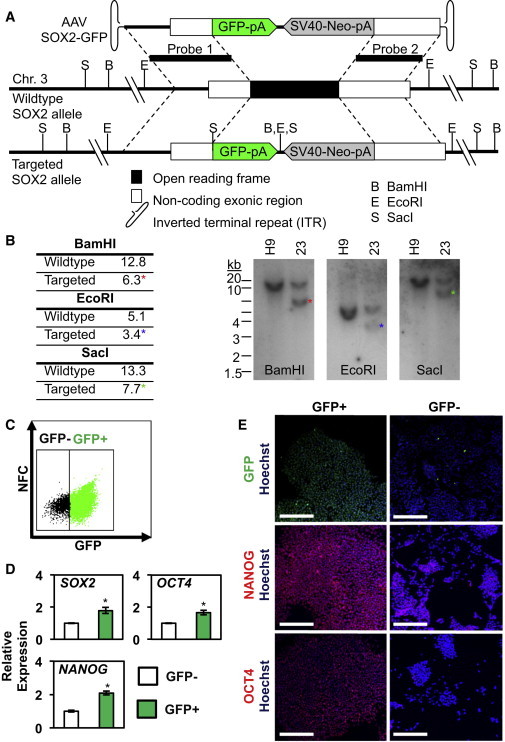
Generation and Characterization of SOX2-GFP Clones (A) Schematic of SOX2-GFP-targeting strategy. The top diagram represents the rAAV targeting vector used for targeting of the *SOX2* locus. The middle diagram depicts the genomic locus of SOX2, a single exon gene, and the bottom diagram illustrates the properly targeted *SOX2* locus. The genetic elements are not displayed to scale. (B) Southern blot using probe-1 (see diagram in [A])-confirmed targeting of the GFP gene to the endogenous *SOX2* locus in hSOX2-23 (23). The bands specific to the targeted allele are not observed in nontargeted wild-type cells (H9). Blots hybridized with probe 2 as well as uncropped blots can be found in Figure S1. (C) Using fluorescence-based cell sorting, undifferentiated hSOX2-23 hESCs were separated on the basis of GFP expression. Wild-type (WT) nonfluorescing H9 hESCs were used as a control to set gates for cell sorting. NFC, nonfluorescent channel. (D) Gene expression analysis by quantitative RT-PCR (qRT-PCR) reveals that pluripotency markers *SOX2*, *OCT4*, and *NANOG* were enriched in the GFP^+^ population. Data represent the mean ± SEM from three independent sorting experiments. Populations were compared using Student’s t test. The asterisk denotes p < 0.05. (E) Representative images of GFP, α-NANOG, and α-OCT4 IF of GFP^+^ and GFP^−^ cells (scale bar represents 200 μm). See also Figures S1 and S2.

Using a recombinant adeno-associated viral (rAAV)-based gene-targeting method, we inserted the gene-encoding GFP into the *SOX2* locus in H9 hESCs (Figure 1A). Proper homologous recombination led to the replacement of the *SOX2* open reading frame with that of GFP and a neomycin selection cassette (SV40-Neo). After infection with rAAV and G418 drug selection, a total of 36 clones were expanded and screened by Southern blotting for homologous recombination events. Among these clones, 26 (72%) were found to carry the GFP-Neo cassette in the *SOX2* locus (Figure S1A available online). No clones in which both *SOX2* alleles were disrupted were isolated. Our subsequent analysis focused on one of these clones, clone 23 (hSOX2-23). We confirmed appropriate gene targeting in this clone using multiple restriction digests followed by Southern blotting (Figures 1B, S1B, and S1C). We did not observe nontargeted insertions of the rAAV sequences, and cells exhibited a normal karyotype (data not shown). Flow cytometry of hSOX2-23 revealed that the majority of the cells expressed GFP (Figure 1C). By comparison, a drug-selected clone, hSOX2-25, which was negative for targeted insertion (Figure S1A), showed no detectable GFP (Figure S2A). Despite only having one copy of *SOX2*, hSOX2-23 had similar levels of *SOX2, OCT4*, and *NANOG* expression as hSOX2-25 and wild-type (WT) hESCs (Figure S2B). Moreover, the percentage of GFP-positive (GFP^+^) cells in hSOX2-23 was constant over more than 20 passages. Immunofluorescence (IF) staining of hSOX2-23 showed that 100% of GFP^+^ cells expressed SOX2 protein (Figure S2C). Additionally, hSOX2-23 colonies had characteristic hESC morphology (Figure S2D) and expressed markers of the undifferentiated state, such as NANOG (Figure S2E). These results show that this rAAV-based gene-targeting method can be used to efficiently disrupt genes by homologous recombination. In addition, the *SOX2*-GFP hESC marker line can be used to monitor SOX2 expression in undifferentiated hESCs.

### SOX2-GFP Marks Undifferentiated hESCs

To investigate whether GFP expression in hSOX2-23 could be used to monitor the differentiation status of hESCs, we performed flow cytometry analysis of hSOX2-23 grown in culture conditions that maintain hESCs in an undifferentiated state. In these conditions, >90% of the cells were GFP^+^ (Figure 1C). Quantitative RT-PCR (qRT-PCR) revealed that expression of *SOX2*, *OCT4*, and *NANOG* was significantly higher in GFP^+^ compared to GFP negative (GFP^−^) cells (Figure 1D), indicating that GFP expression marked undifferentiated cells. To determine if GFP expression could be used to remove differentiating cells from pluripotent hESC cultures, we cultured purified GFP^+^ and GFP^−^ cells in conditions that support undifferentiated growth for hESCs. The GFP^+^ cells grew as compact colonies characteristic of the undifferentiated state, whereas the GFP^−^ cells had a fibroblast-like morphology typical of differentiating hESCs (Figure S2F). Moreover, cultured GFP^+^ cells maintained high GFP expression, whereas cultured GFP^−^ cells failed to express detectable levels of GFP (Figure 1E). Finally, IF staining revealed that cultured GFP^+^ cells maintained high NANOG and OCT4 expression, whereas cultured GFP^−^ cells showed little NANOG or OCT4 expression (Figure 1E). These results suggest that the SOX2-GFP marker can be used to monitor the undifferentiated state of hESCs.

### Dynamics of SOX2-GFP Expression during Neural Differentiation

**Figure 2:**
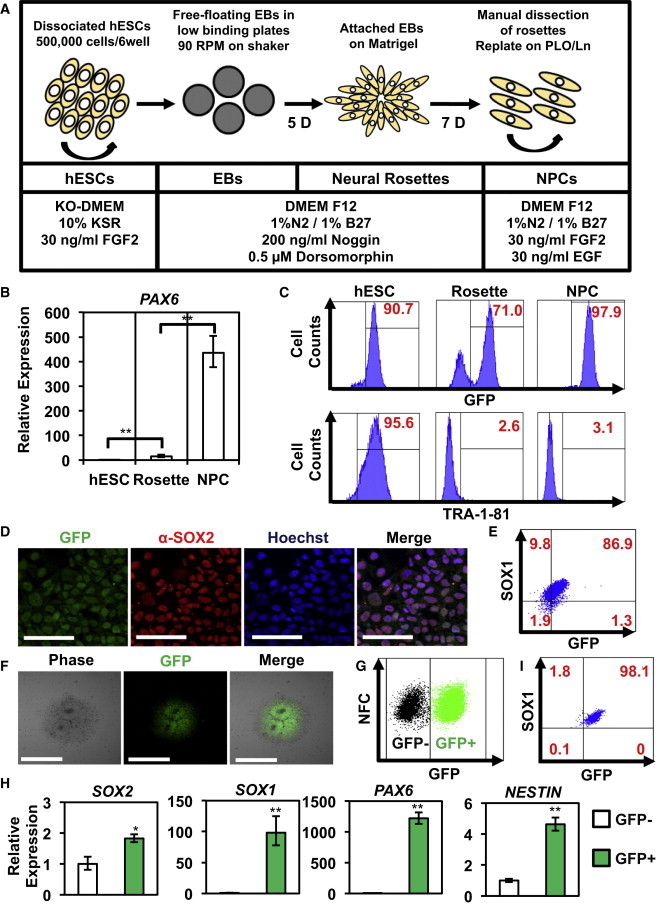
Differentiation of SOX2-GFP hESC to Neurectoderm Lineages (A) Outline of protocol for differentiation of hESCs to NPCs. The soluble factors, substrate, and culture media at each stage are indicated. KO, knockout; KSR, KnockOut serum replacement. (B) Gene expression analysis for the neurectoderm marker *PAX6* during hESC differentiation to rosettes and NPCs (n = 3 independent experiments; error bars represent ± SEM; ^∗∗^p < 0.01). (C) Flow cytometry analysis of TRA1-81 and GFP during NPC differentiation. Isotype controls used are listed in Table S4. (D) IF analysis of GFP and SOX2 showed colocalization in NPCs (scale bar represents 100 μm). (E) Flow cytometry analysis of SOX1 expression in SOX2-GFP NPCs. SOX1 shows high coexpression with GFP. Isotype controls used are listed in Table S4. (F) IF of SOX2-GFP hESCs differentiated to neural rosettes (scale bar represents 500 μm). (G) SOX2-GFP neural rosette cells were sorted on the basis of GFP expression. WT H9 rosettes were used as a control to set gates for cell sorting. (H) Gene expression analysis of sorted GFP^+^ and GFP^−^ cells showed high expression of NPC markers *SOX2*, *SOX1*, *NESTIN*, and *PAX6* in GFP^+^ cells. Data represent the mean ± SEM from three independent sorting experiments. Populations were compared using Student’s t test. The asterisk denotes p < 0.05 and double asterisks denote p < 0.01. (I) Flow cytometry analysis of SOX1 expression in FACS-purified GFP^+^ cells. Replated GFP^+^ cells maintained high expression of GFP and SOX1.

In addition to being a master regulator of hPSCs, SOX2 is a marker of multipotent NPCs and is necessary for their maintenance in the nervous system (Ellis et al., 2004). To assess regulation of the SOX2-GFP marker during neurectoderm differentiation, we developed a serum-free differentiation protocol based on previously published methods (Figure 2A; Chambers et al., 2009; Li et al., 2011). In brief, NPCs were manually picked from embryoid body-derived rosettes, dissociated, replated, and maintained as proliferative cells in the presence of fibroblast growth factor 2 (FGF2) and epidermal growth factor (EGF) (Shin et al., 2006). Expression of SOX2 and the neural-specific marker *PAX6* peaked upon NPC formation (Figure 2B). Flow cytometry confirmed the progressive loss of the pluripotency marker TRA-1-81 as hESCs differentiated to rosettes and NPCs (Figure 2C). Concurrently, GFP expression declined upon differentiation to the rosette stage and then re-emerged in NPCs (Figure 2C). This pattern of GFP expression is consistent with previous studies (Chambers et al., 2009). IF of hSOX2-23 NPCs revealed that 100% of GFP^+^ NPCs were SOX2^+^ (Figure 2D). Additionally, a high percentage of GFP^+^ NPCs coexpressed the NPC marker SOX1 as monitored by flow cytometry (Figure 2E). Together, these results demonstrate that SOX2-GFP expression can be used to monitor neural differentiation of hESCs.

### Isolation of SOX2-GFP^+^ NPCs from Neural Rosettes

Fluorescence analysis of rosette stage cultures revealed that GFP expression was isolated to the neuroepithelial-like rosette structures that are manually dissected to obtain NPCs (Elkabetz et al., 2008; Figure 2F). To investigate if GFP expression could allow for the isolation of NPCs without manual dissection, we dissociated rosette-stage cultures into single cells and isolated GFP^+^ cells using fluorescence-based cell sorting (Figure 2G). Gene expression analysis of GFP^+^ and GFP^−^ cell populations by qRT-PCR revealed that the GFP^+^ rosette stage cells expressed higher amounts of the NPC markers *SOX1*, *SOX2*, *PAX6*, and *NESTIN* than the GFP^−^ rosette stage cells (Figure 2H). Subsequent culture of GFP^+^ rosette stage cells revealed that these cells maintained high expression of GFP and the NPC marker SOX1 (Figure 2I). Thus, SOX2-GFP expression can be used to isolate NPCs from rosette stage cultures.

### SOX2-GFP Marks the Anterior Foregut Endodermal Progeny of Differentiating hESCs

**Figure 3:**
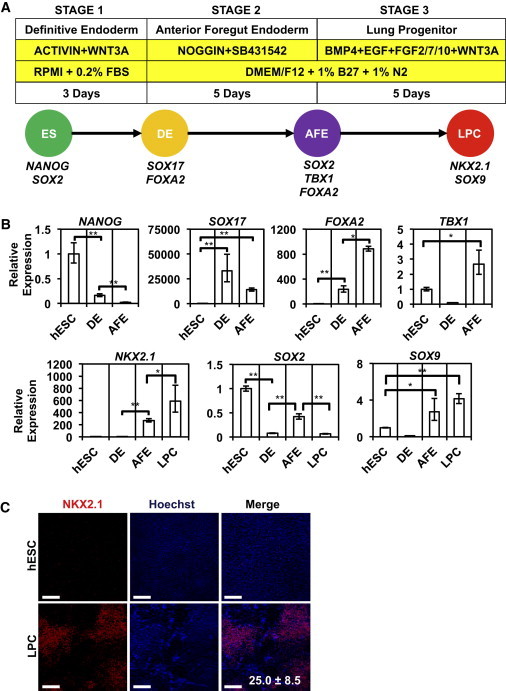
Differentiation of hESCs to Anterior Foregut and Lung Endoderm (A) Outline of protocol for differentiation of hESCs to anterior foregut and lung progenitor cells. The soluble factors and culture media at each stage are shown. (B) Gene expression analysis of markers of undifferentiated hESCs (*NANOG*, *SOX2*), definitive endoderm (DE; *SOX17*), anterior foregut endoderm (AFE; *SOX2*, *FOXA2*, *TBX1*), and lung progenitor cell (LPC; *NKX2.1*, *SOX9*; n = 3 independent experiments; error bars represent ± S.E.M; ^∗^p < 0.05; ^∗∗^p < 0.01). (C) IF for NKX2.1 on day 13 LPC cultures (mean ± SD; scale bar represents 200 μm). See also Figure S3.

*SOX2* is expressed in the developing AFE, with the highest levels in the future esophagus, trachea, and lung (Que et al., 2007). To investigate if the hSOX2-23 line could be used to isolate cells with an AFE identity from differentiating hESCs, we used a modified version of previously published protocols (Figure 3A; Green et al., 2011; Longmire et al., 2012; Mou et al., 2012). To generate definitive endoderm (DE), the precursor cell population for AFE, hSOX2-23s were treated with Activin A and Wnt3a (stage 1). Subsequent differentiation to AFE was achieved through addition of bone morphogenetic protein (BMP) antagonists noggin and SB431542 (stage 2). Maturation to a lung progenitor cell (LPC) phenotype was achieved through addition of BMP4, FGF2, and Wnt3a. At stage 1, expression of the DE marker *SOX17* peaked while expression of the pluripotency marker *NANOG* declined (Figure 3B). Similarly, at stage 2, we observed high expression levels of AFE markers *FOXA2* and *TBX1* (Figure 3B). In contrast, expression of the posterior foregut endoderm (PFE) markers *HNF6* and *PDX1* was not detectable (Figure S3A). *SOX2*, as well as *TBX1*, expression re-emerged during differentiation to AFE (Figure 3B), but not to PFE (Figure S3B), suggesting the SOX2-GFP reporter line can be employed to isolate cells with properties associated with AFE. Furthermore, LPC markers *NKX2.1* and *SOX9* showed high levels of expression at stage 3 (Figure 3B). Consistent with previous publications, this protocol yielded approximately 25% NKX2.1-positive (NKX2.1^+^) LPCs (Figure 3C).

**Figure 4:**
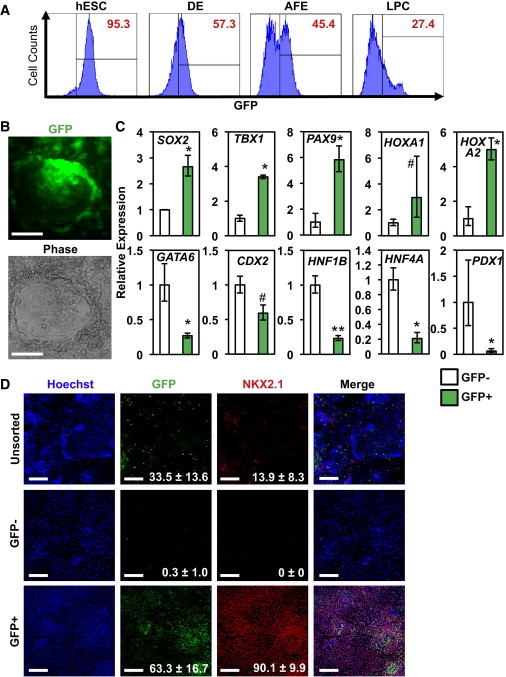
Characterization of SOX2-GFP Reporter hESCs Differentiation to AFE (A) Flow cytometry analysis of SOX2-GFP dynamics during hESC differentiation to AFE and LPC. (B) IF analysis of day 8 SOX2-GFP AFE cultures (scale bar represents 200 μm). (C) Gene expression analysis showed that AFE markers (*SOX2*, *TBX1*, *PAX9*, *HOXA1*, *HOXA2*) were highly enriched in GFP^+^ cells. The expression levels of markers of the posterior foregut endoderm (PFE; *HNF1B*, *HNF4A*, *GATA6*, *CDX2*, *PDX1*) were higher in GFP^−^ cells (n = 3 independent experiments; error bars represent ± SEM). (D) IF analysis of GFP^+^, GFP^−^, or unsorted control cells that were purified using fluorescence-based cell sorting at day 8 of differentiation, replated, and differentiated to LPCs. Expression of GFP and the LPC marker NKX2.1 was enriched in in-vitro-differentiated GFP^+^ cells versus GFP^−^ or unsorted control cells (mean ± SD; scale bar represents 200 μm).

Next, we tested whether GFP expression in hSOX2-23 hESCs could be used to monitor anterior foregut differentiation and to purify AFE progeny. After an initial decrease in GFP expression as cells exited the pluripotent state and differentiated toward DE, a GFP^+^ cell population re-emerged and expanded during the AFE stage (Figure 4A). Upon subsequent differentiation to LPC, GFP expression disappeared. This temporal pattern of GFP expression was consistent with our analysis of *SOX2* gene expression. Differentiation of hSOX2-23 into AFE yielded densely packed GFP^+^ cells, often surrounding an empty lumen-like cavity (Figure 4B). qRT-PCR analysis revealed that expression of the AFE markers *SOX2*, *TBX1*, *PAX9*, *HOXA1*, and *HOXA2* was highly enriched in the GFP^+^ population, whereas the PFE markers *HNF1B*, *HNF4A*, *GATA6*, *CDX2*, and *PDX1* were enriched in the GFP^−^ population (Figure 4C). To test the potential of these cells to develop into LPCs, sorted GFP^+^ and GFP^−^ AFE cells and unsorted control AFE cells were replated and differentiated to LPCs using previously described methods (Green et al., 2011; Longmire et al., 2012; Mou et al., 2012). IF analysis for NKX2.1, the earliest marker of LPCs distinguishing it from the remainder of the AFE (Fagman et al., 2011; Que et al., 2009), revealed that >90% of GFP^+^ cells differentiated into NKX2.1^+^ lung endoderm (Figure 4D). In contrast, the unsorted AFE population or the GFP^−^ populations generated significantly fewer SOX2^+^ and NKX2.1^+^ cells (Figure 4D). Together, these results demonstrate that GFP^+^ cells exhibit properties of AFE and are capable of differentiating in vitro into derivatives of AFE, including NKX2.1^+^ LPCs.

### Genome-wide Analysis of SOX2-GFP Reporter-Expressing Anterior Foregut Endoderm Cells

**Figure 5:**
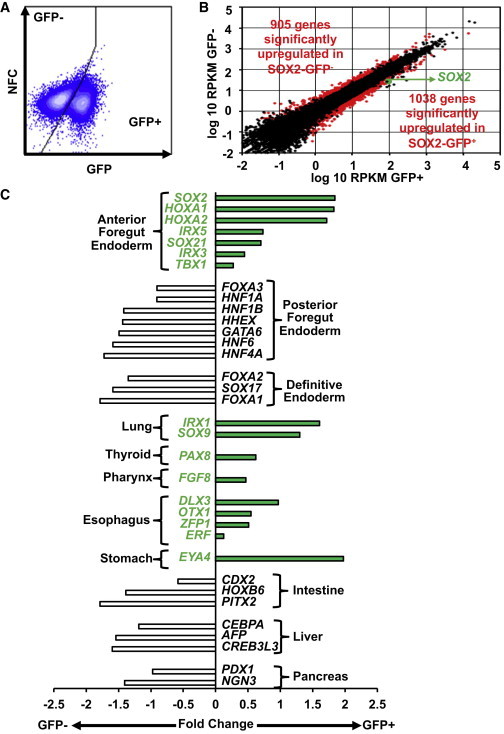
Genome-wide Expression Analysis of SOX2-GFP AFE (A) Day 8 AFE SOX2-GFP cells were separated by fluorescence-based cell sorting on the basis of GFP expression. (B) Scatter plot of log_10_ RPKM in GFP^+^ and GFP^−^ day 8 AFE cells. Genes with a statistically significant difference are shown in red. (C) Selection of differentially expressed genes highlighting differences in gene expression patterns related to patterning and differentiation of the foregut endoderm. See also Table S1.

To define a global gene expression signature of AFE, we performed whole transcriptome sequencing (RNA sequencing [RNA-seq]) of sorted GFP^+^ and GFP^−^ cells from differentiated AFE cultures (Figure 5A and Table S1). We identified 1,943 genes with differential expression between these two cell populations, with the expression of 1,038 genes elevated in the GFP^+^ population and 905 genes elevated in the GFP^−^ population (Figure 5B). This signature included genes involved in signaling pathways (Wnt, FGF, Notch, BMP, and RA signaling) known to play a role in the patterning of the foregut endoderm. Moreover, this genetic signature included genes known to define developing AFE and PFE (Figure 5C). Specifically, expression of AFE markers *SOX2*, *HOXA1*, *HOXA2*, and *IRX5* was highly enriched in the GFP^+^ population (Figure 5C). Conversely, expression of PFE markers *HNF1A*, *HNF1B*, *HNF6*, and *GATA6* as well as the DE markers *SOX17* and *FOXA1* were increased in the GFP^−^ cells (Figure 5C). Early markers of tissues derived from AFE, such as the lung (*IRX1* and *SOX9*), thyroid (*PAX8*), pharynx (*FGF8*), esophagus (*DLX3* and *OTX1*), and stomach (*EYA4*), showed higher levels of expression in GFP^+^ cells (Figure 5C). In contrast, expression of genes associated with tissues derived from PFE, such as intestine (*CDX2*), liver (*AFP*), and pancreas (*PDX1* and *NGN3*), were lower in GFP^+^ cells (Figure 5C). Collectively, this RNA-seq analysis suggests that GFP^+^ cells isolated from differentiating cultures are enriched for cells with an AFE gene expression profile.

### Isolation of AFE Using Cell Surface Markers

**Figure 6:**
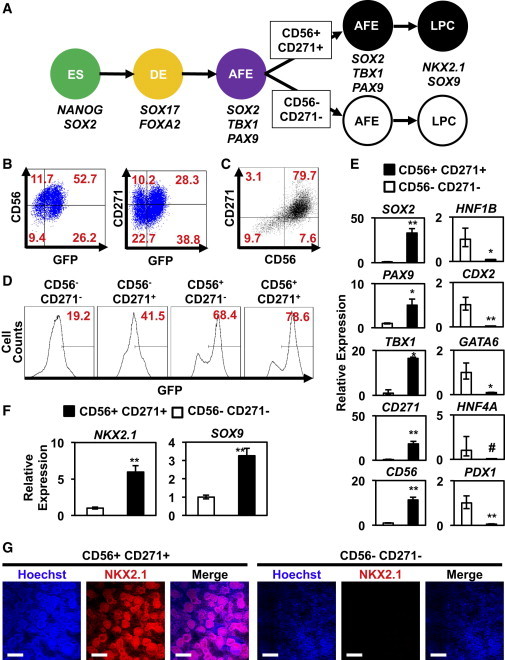
Cell Surface Markers Expressed in hESC-Derived AFE (A) HESC-differentiated AFE cells were sorted based on levels of CD56 and CD271 expression. Double-positive CD56^+^CD271^+^ and double-negative CD56^−^CD271^−^ cells were replated and further differentiated in vitro to LPCs. (B) Flow cytometry analysis demonstrated that CD56 and CD271 expression correlates with GFP expression in day 8 AFE SOX2-GFP cells. (C) HESC-differentiated AFE cells were sorted on the basis of CD56 and CD271. (D) Flow cytometry analysis shows that GFP expression is highest in double-positive CD56^+^CD271^+^ compared to single-positive CD56^+^CD271^−^ or CD56^−^CD271^+^ cells or double-negative CD56^−^CD271^−^ cells. (E) Gene expression analysis reveals that the expression of the AFE markers *SOX2*, *TBX1*, and *PAX9* were highly enriched in the CD56^+^CD271^+^ cells. As expected, expression of CD56 and CD271 was enriched in CD56^+^CD271^+^ cells. Conversely, expression of the PFE markers *GATA6*, *HNF1B*, *HNF4A*, *CDX2*, and *PDX1* were enriched in CD56^−^CD271^−^ cells. (F) Expression of LPC markers *NKX2.1* and *SOX9* was enriched in in-vitro-differentiated CD56^+^CD271^+^ cells. Data represent the mean ± SEM from three independent sorting experiments. Populations were compared using Student’s t test. The number sign denotes p > 0.05, asterisk denotes p < 0.05, and double asterisks denote p < 0.01. (G) IF analysis of CD56^+^CD271^+^ and CD56^−^CD271^−^ cells that were purified by fluorescence-based cell sorting at day 8, replated, and differentiated to LPCs. Expression of the LPC marker NKX2.1 was enriched in in-vitro-differentiated CD56^+^CD271^+^ cells versus CD56^−^CD271^−^ cells (scale bar represents 200 μm). See also Figures S4 and Table S2.

To develop a cell surface marker “signature” for SOX2^+^ AFE cells, we mined our RNA-seq data for genes encoding transmembrane proteins with differential expression levels between GFP^+^ and GFP^−^ cells (Table S2). qRT-PCR confirmed that several genes encoding cell surface markers were differentially expressed in the GFP^+^ and GFP^−^ populations (Figures S4A and 4B). Flow cytometry with antibodies directed against these cell surface markers revealed that staining of CD56 (neural cell adhesion molecule [NCAM]) and CD271 (nerve growth factor receptor [NGFR]) correlated with GFP expression in day 8 AFE cells (Figures 6A and 6B, and S4C). Consistent with the RNA-seq data, qRT-PCR analysis confirmed that transcripts for both CD56 and CD271 were enriched in the GFP^+^ populations (Figure S4B). Similarly, gene expression analysis of cells at various stages of differentiation revealed that CD56 and CD271 expression peaked at AFE (Figure S4D).

Using fluorescence-based cell sorting of day 8 AFE cultures with antibodies to CD56 and CD271 (Figures 6A and 6C), we demonstrated that double-positive CD56^+^CD271^+^ cells expressed higher levels of GFP than single-positive CD56^+^CD271^−^ or CD56^−^CD271^+^ cells or double-negative CD56^−^CD271^−^ cells (Figure 6D). When AFE cells differentiated from WT H9 hESCs were sorted for these cell surface markers, expression of AFE markers *SOX2*, *TBX1*, and *PAX9* was increased in double-positive cells compared with double-negative cells (Figure 6E). Conversely, expression of PFE markers *GATA6*, *HNF1B*, *HNF4A*, *CDX2*, and *PDX1* was higher in double-negative cells compared with double-positive cells (Figure 6E). To investigate if double-positive CD56^+^CD271^+^ cells were capable of differentiating into more mature lung progeny, as assessed by *NKX2.1* expression, we replated CD56^+^CD271^+^ and CD56^−^CD271^−^ cells after cell sorting and differentiated them to LPCs. Gene expression analysis revealed that expression of the LPC markers *NKX2.1* and *SOX9* was enriched in the CD56^+^CD271^+^ population relative to the CD56^−^CD271^−^ (Figure 6F). Additionally, IF analysis for NKX2.1 revealed that a higher percentage of the CD56^+^CD271^+^ cells differentiated into NKX2.1^+^ lung endoderm compared to the CD56^−^CD271^−^ cells (Figure 6G). Interestingly, cells with highest NKX2.1 expression were clustered with bright staining along the edges, an organization reminiscent of an epithelial cell population as may be expected for lung epithelial precursors. These data demonstrate that cell enrichment strategies for CD56 and CD271 significantly increase the percentage of cells with AFE gene expression patterns from differentiated hESC cultures.

**Figure 7:**
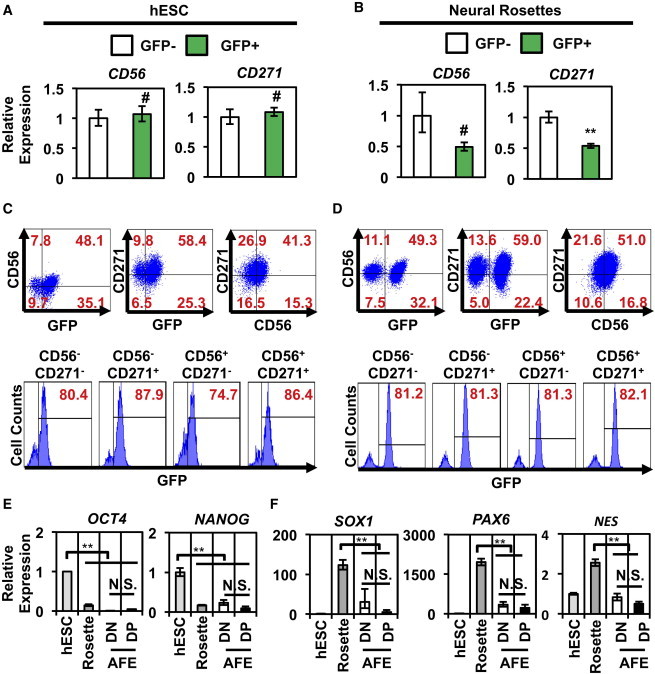
CD56 and CD271 Do Not Mark a SOX2^+^ hESC or Neural Population Gene expression analysis of GFP^+^ and GFP^−^ undifferentiated hESCs (A) and neural rosette cells (B) shows that expression of *CD56* and *CD271* is not enriched in GFP^+^ or GFP^−^ cell populations (n = 3 independent experiments; error bars represent ± SEM, #p > 0.05). Flow cytometry analysis demonstrates that CD56 and CD271 do not correlate with GFP expression in undifferentiated hESCs (C) and neural rosettes (D). Double-positive CD56^+^CD271^+^ AFE cells are not enriched for hESC- (E) or neural- (F) related markers (n = 3 independent experiments; error bars represent ± SEM; ^∗∗^p < 0.01. DP, double-positive CD56^+^CD271^+^ AFE; DN, double-negative CD56^−^CD271^−^ AFE; NS, no statistically significant difference).

Although our analysis suggested that CD56 and CD271 marked SOX2^+^ AFE cells, it was unclear if these cell surface markers specified SOX2^+^ cells in undifferentiated hESC or neurectoderm cultures. Gene expression analysis revealed that neither *CD56* nor *CD271* expression was enriched in GFP^+^ hESCs (Figure 7A) or neural rosette cells (Figure 7B). Flow cytometry revealed that neither CD56 nor CD271 correlated with GFP expression in hESCs (Figure 7C) or neural rosette cells (Figure 7D). Because SOX2 is also expressed in undifferentiated hESCs and neurectoderm cells, we wanted to confirm that we were not enriching these rare cell types in our CD56^+^CD271^+^ AFE cultures. To that end, there was little expression of the pluripotency markers *OCT4* and *NANOG* in CD56^+^CD271^+^ AFE cells when compared to undifferentiated hESCs (Figure 7E). Additionally, there was no difference in expression of these genes between the CD56^+^CD271^+^ and CD56^−^CD271^−^ AFE cells. Furthermore, expression of the neurectoderm markers *SOX1*, *PAX6*, and *NES* was significantly lower in CD56^+^CD271^+^ AFE cells compared to neural rosette cells (Figure 7F). Finally, there was no difference in expression of these genes among the CD56^+^CD271^+^ and CD56^−^CD271^−^ AFE cells. Collectively, these studies suggest that CD56 and CD271 expression correlates only with the SOX2^+^ AFE cell population.

## Discussion

In this work, we employed a human SOX2-GFP reporter cell line to characterize distinct cell populations in which *SOX2* is known to be expressed, including undifferentiated hPSCs, NPCs, and anterior foregut endodermal cells (AFEs). We showed that this reporter line can be used to monitor the differentiation status of cells, isolate and purify distinct cell populations, and identify genes with expression patterns associated with these distinct cell populations. This approach is particularly valuable for the design and development of protocols for the directed differentiation of hPSCs into cell populations suitable for transplantation studies, disease modeling, and drug screening.

### Gene Targeting Using AAV

Gene targeting in hPSCs has met many challenges, and to date, methods for homologous recombination (HR) in hPSCs are not as commonplace as in mouse embryonic stem cells (mESCs). The reasons for differences in gene targeting between mESCs and hPSCs remain poorly understood. It has been suggested that mESCs represent an early “naive” developmental stage akin to the inner cell mass of the blastocyst, whereas hPSCs represent a later “primed” developmental state that resembles the epiblast (Nichols and Smith, 2009), and that this difference accounts for the differences observed in transgenesis and HR (Buecker et al., 2010). In fact, Buecker et al. showed that, in hPSCs that had been genetically manipulated to obtain a naive mESC-like state, HR targeting efficiencies approached those typically observed in mESCs (Buecker et al., 2010). However, conversion of hPSCs to a naive state with biological characteristics similar to mESCs remains technically challenging (Hanna et al., 2010).

Based on several previous publications (Khan et al., 2010, 2011), we explored the utility of adeno-associated virus (AAV) as a method to improve gene targeting efficiencies in hPSCs. For *SOX2*, a gene that is highly expressed in undifferentiated hPSCs, gene targeting rates were greater than 70%. Similar targeting efficiencies in hPSCs using AAV have been reported by others (Asuri et al., 2012; Khan et al., 2010, 2011; Smith-Arica et al., 2003), indicating that AAV offers a highly efficient and robust approach to target genes for HR in hPSCs.

Currently, conventional methods for gene targeting utilize standard transduction methods, such as electroporation, to introduce linearized DNA constructs with homology arms of 3–5 kb flanking positive (e.g., neomycin or hygromycin) and negative (ganciclovir) selection cassettes (Mansour et al., 1988). These approaches are extremely inefficient, with targeting efficiencies varying between <0.1% and 5%. An alternative method to improve gene modification efficiencies involves the introduction of site-specific, double-stranded breaks into the genome using zinc finger nucleases (Davis and Stokoe, 2010; Zou et al., 2009), transcription activator-like effector nucleases (Hockemeyer et al., 2011), or the RNA-guided CRISPR-Cas system (Cong et al., 2013; Hou et al., 2013; Mali et al., 2013). Whereas these approaches are promising, site-directed specificity has been difficult to control and off-target cleavage events are common (Cradick et al., 2013; Radecke et al., 2010). In addition, bacterial artificial chromosomes (BACs) have been successfully used for site-specific targeting in hPSCs at efficiencies of up to 25% (Song et al., 2010); however, the use of BACs is technically challenging due to complex cloning methods.

In contrast to these methods, AAV offers features that make it an attractive alternative means for gene targeting. First, the AAV genome is relatively compact (∼4.8 kb) and genetic engineering is accordingly straightforward. Aside from two flanking palindromic inverted terminal repeats, the entire genome can be engineered to contain the desired genetic elements, including drug selection cassettes, reporter genes, and homology arms to promote HR. Second, AAV is a single-stranded DNA virus and, upon infection and entry into the cell, this single-stranded piece of DNA provides an ideal substrate for the endogenous DNA repair machinery, thereby significantly increasing gene-targeting efficiencies. Third, AAV rarely integrates itself nonspecifically into the genome and it consequently has become an attractive system to create viral vectors for gene therapy.

### SOX2, a Regulator of Pluripotency

Along with *OCT4* and *NANOG*, *SOX2* is one of the master regulators of the pluripotent state in hPSCs (Rizzino, 2009). However, analysis of *SOX2* expression in hPSCs relies on the fixation of cells, which limits their use in subsequent molecular and biological studies. We were able to use our SOX2-GFP reporter line to detect and enrich for SOX2 expression in live hPSC cultures. Consistent with previous reports that describe hPSCs as heterogeneous cultures with varying levels of expression of pluripotency-associated genes (Stewart et al., 2006), we observed varying levels of SOX2-GFP expression in our hPSC cultures. Purification and analysis of these cells revealed higher expression levels of pluripotency-associated genes in SOX2^+^ versus SOX2^−^ cells. Moreover, subsequent culture of purified SOX2^+^ and SOX2^−^ cells revealed that they maintained distinct developmental states.

### SOX2, a Regulator of NPC Multipotency

NPCs derived from hPSCs offer a unique model system to study neural development and are a possible source of cells to treat a variety of neurodegenerative disorders. In the adult brain, SOX2 functions to maintain the multipotent state of endogenous NPCs (Graham et al., 2003). Further, SOX2 is a marker of multipotent NPCs derived from hPSCs (Chambers et al., 2009; Li et al., 2011). Consistent with these studies, we were able to use SOX2-GFP reporter expression to isolate a homogeneous population of SOX2^+^ NPCs from heterogeneously differentiating cultures. Moreover, we demonstrated that these cells were enriched for neural markers and maintained high expression of NPC markers over subsequent passages. These SOX2^+^ NPCs will be useful for future applications, such as neural transplantation, genetic profiling, or epigenetic analysis.

### SOX2, a Marker of Gut Tube Patterning

Cells derived from AFE, including those comprising the lung, trachea, and thyroid, are of significant interest for many regenerative medicine and disease-modeling purposes. SOX2 has been implicated in regulating the patterning of the foregut endoderm along the anterior-posterior axis and specifying AFE (Que et al., 2007). Using our SOX2-GFP reporter line, we were able to monitor foregut endoderm differentiation and use flow cytometry to isolate a pure SOX2^+^ AFE population from differentiating cultures and perform subsequent genetic and developmental studies. Using RNA-seq, we were able to identify a global gene expression signature that defines SOX2^+^ AFE cells in heterogeneously differentiating hPSC cultures. Because AFE exists only transiently during in vivo development, our SOX2 reporter line allows for the in vitro study of a developmental stage that is difficult to analyze in vivo. Finally, we demonstrated that subsequent in vitro differentiation of sorted SOX2^+^ cells led to the generation of cells that uniformly express NKX2.1, a transcriptional regulator of lung and thyroid development. Together, this cellular platform will be useful for future studies examining the developmental and genetic programs that contribute to foregut, lung, and thyroid development.

Flow-cytometry-based purification of intermediate progenitor cell populations of differentiating hPSCs followed by subsequent differentiation is an alternative approach for generating highly enriched and well-defined mature cell populations required for cell-based therapies and disease modeling (McKnight et al., 2010). Recently, transgenic cell marking combined with genome-wide expression profiling and flow cytometry have been used to develop flow-cytometry-based strategies for the purification of DE, immature cardiomyocytes, and pancreatic endoderm (Dubois et al., 2011; Kelly et al., 2011; Wang et al., 2011). However, flow-cytometry-based strategies have not yet been developed for the purification of AFE. Using our genome-wide expression analysis of reporter-expressing AFE cells, we identified two cell surface markers, CD56 (also known as NCAM) and CD271 (also known as NGFR), that permitted the isolation of SOX2^+^ AFE cells. Although their names imply neural expression (and hence ectodermal origin), CD56/NCAM and CD271/NGFR are not completely restricted to the derivatives of the ectodermal germ layer. For example, these cell surface markers also define cells of the mesodermal (Evseenko et al., 2010), mesenchymal (Saliem et al., 2012), and other nonneural lineages (Yuan et al., 2011). In the future, this cell surface panel will allow for the prospective isolation and study of pure AFE cells from potentially any hPSC line.

In conclusion, we have developed a cell-based tool that will allow for the study of SOX2^+^ cells, not only in pluripotent hPSCs, but also in various endodermal and neural-related cell types. Furthermore, this reporter cell line will enable high-throughput screening approaches to identify secreted factors or small molecules that promote endodermal or neural differentiation of hPSCs. Finally, because SOX2 is one of the few regulatory genes expressed in both pluripotent and differentiated cells, future genetic and epigenetic analysis of the SOX2^+^ cell populations will allow for the identification of common mechanisms that control hPSC pluripotency and differentiation.

## Experimental Procedures

### Cells and Culture Conditions

Media compositions and sources for all cell lines are listed in the Supplemental Information section. All hESC cultures were supplemented with 30 ng/ml FGF2. Mouse embryonic fibroblast-conditioned medium (MEF-CM) was produced by culturing MEFs in hESC medium for 24 hr followed by sterile filtering. Cells were routinely passaged with Accutase, washed, and replated at a density 4.25 × 10^4^/cm^2^. All work with hESCs was reviewed and approved by the University of California at San Diego (UCSD) Stem Cell Research Oversight Committee, project numbers 100210ZX and 090807ZX.

### AAV Production and Gene Targeting

The design and construction of the *SOX2*-targeting vector is described in the Supplemental Experimental Procedures. Supernatants carrying infectious AAV particles were produced as previously described (Hirata et al., 2002) with a detailed protocol available here: http://vectorcore.salk.edu/protocols/AAV Production Protocol.doc. H9 cells on Matrigel in MEF-CM were infected with the SOX2-GFP AAV-2 supernatants at approximately 10^10^ genome copies. After 24 hr, virus was removed and G418 (50 μg/ml) was applied for 2 weeks. After 2 weeks, colonies were manually picked and transferred to fresh MEF feeder cells in 96-well plates. Genomic DNA extracted from G418^R^ clones was analyzed using Southern blot analysis with probes to the left (probe 1) and right (probe 2) homology arms.

### Neural Differentiation and NPC Culture

Methods to derive and passage NPCs are described in detail in the Supplemental Experimental Procedures. Briefly, embryoid bodies formed over 5 days in the presence of 50 ng/ml recombinant mouse noggin (R&D Systems) and 0.5 μM Dorsomorphin (Tocris Bioscience) were cultured in neural induction media. After 7 days, neural rosettes were isolated, dissociated into single cells, and plated onto poly-L-ornithine (10 μg/ml) and mouse laminin (5 μg/ml)-coated dishes in neural induction media with 10 ng/ml mouse FGF2 and 10 ng/ml mouse EGF2.

### Endodermal Differentiation

Methods to differentiate hESCs to endodermal derivatives are described in detail in the Supplemental Experimental Procedures. Briefly, to generate DE, hESCs were cultured for 3 days in 100 ng/ml recombinant human Activin A with the first day supplemented with 30 ng/ml Wnt3a (Willert et al., 2003). For differentiation of AFE, DE cells were treated for 5 days with 200 ng/ml noggin and 10 μM SB-431542. For differentiation to LPCs, AFE cells were treated for 5 days with 100 ng/ml Wnt3a, 10 ng/ml mouse keratinocyte growth factor (KGF/FGF7), 100 ng/ml mouse FGF2, 10 ng/ml mouse BMP4, 10 ng/ml mouse FGF10, and 10 ng/ml EGF. For differentiation to PFE, DE cells were cultured in 50 ng/ml KGF for 3 days and then in 50 ng/ml noggin, 0.25 μM 3-keto-N-(aminoethyl-aminocaproyl-dihydrocinnamoyl)cyclopamine, and 2 μM retinoic acid for 3 days.

### qRT-PCR

RNA was isolated using TRIzol and reverse transcription was performed by means of qScript cDNA Supermix. qRT-PCR was carried out using TaqMan probes (Table S3) and TaqMan Fast Universal PCR Master Mix on a 7900HT Real Time PCR machine. Gene expression was normalized to 18S rRNA levels. All experiments were performed with three technical replicates.

### IF and FC

Detailed protocols for IF and flow cytometry (FC) are provided in the Supplemental Information section. For IF, cultures were in 4% (w/v) paraformaldehyde, permeabilized with 0.2% (v/v) Triton X-100, washed, and incubated overnight in primary antibody. Secondary antibodies were incubated 1 hr. All antibodies and dilutions are listed in Table S4. Nucleic acids were stained for DNA with Hoechst 33342 (2 μg/ml). Cells were imaged on an Olympus Fluoview 1000. Image quantation was performed by counting a minimum of nine fields at 20× magnification. For FC, cells were dissociated into single cells with Accutase, washed with fluorescence-activated cell sorting (FACS) buffer, resuspended at 5 × 10^6^ cells per 100 μl, stained with indicated antibodies (Table S4), and analyzed and sorted with a FACSCanto or FACSAria2 (BD Biosciences). FC data were analyzed with FACSDiva software. For replating experiments, cells were stained with appropriate antibodies, sorted in FACS buffer, and replated with 10 nM Y27632. Isotype negative controls are listed in Table S4. For sorting experiments in which cells were separated on the basis of GFP expression, wild-type nonfluorescing cells were used as a negative control.

### High-Throughput RNA-Seq

RNA-seq of RNA from SOX2-GFP^+^ and SOX2-GFP^−^ AFE cells was performed as described in the Supplemental Information section, and differential gene expression analysis was performed with TopHat and Cufflinks (Trapnell et al., 2012, 2013). Reads per kilobase of exon per million mapped reads (RPKM) were calculated for each gene and used as an estimate of expression levels.

## Supplemental Information

Document S1. Supplemental Experimental Procedures, Figures S1–S4, and Tables S3 and S4

Table S1. RNA-Seq Data of GFP^+^ and GFP− Day 8 AFE Cells, Related to Figure 5

Table S2. RNA-Seq Data of Cell Surface Markers Differentially Expressed in GFP^+^ and GFP− Day 8 AFE Cells, Related to Figure 6
