# Supplementary material for: Analysis of SOX2-Expressing Cell Populations Derived from Human Pluripotent Stem Cells
Source: Stem Cell Reports. 2013 Oct 31;1(5):464–78. doi: 10.1016/j.stemcr.2013.09.005 (PMC3841266; doi:10.1016/j.stemcr.2013.09.005)
Supplement: Document S1. Supplemental Experimental Procedures, Figures S1–S4, and Tables S3 and S4 [file mmc1.pdf]

Stem Cell Reports, Volume *1*

Supplemental Information

Analysis of SOX2-Expressing Cell Populations

Derived from Human Pluripotent Stem Cells

David A. Brafman, Noel Moya, Stephanie Soltero, Thomas Fellner, Megan Robinson, Zoë Vomberg McMillen, Terry Gaasterland, and Karl Willert

INVENTORY OF SUPPLEMENTAL INFORMATION

Provided with this article are the following supplemental items.

Supplemental Experimental Procedures

A detailed summary of all experimental procedures used in this study. An abbreviated version of these methods is provided in the Main Text.

Figures S1-4

Figure S1: This figure relates to Figure 1 and shows images of the Southern blots used to determine the approximate targeting efficiency of 72%. A. Southern blot analysis of all expanded G418 selected clones. B. Uncropped image of Southern blot shown in Figure 1B. C. Summary of expected fragment sizes in Southern blots.

Figure S2: This figure relates to Figure 1 and shows GFP expression of both a targeted and a non-targeted line (A). It also shows standard characterizations of the reporter line hSOX2-23, including expression of pluripotency genes *SOX2*, *OCT4,* and *NANOG* (B, n=3, error bars = ± S.E.M, abbreviations: W.T.= wild-type H9 hESCs, N.S.= no statistically significant difference), co-expression of GFP and SOX2 (C; scale bar = 100 µm), a phase contrast image to illustrate the characteristic hPSC morphology (D; scale bar = 500 µm), and protein expression of the pluripotency gene *NANOG* (E; scale bar = 100 µm). It also shows the morphology of flow cytometry sorted GFP positive and GFP negative hESCs (F; scale bar = 200 µm).

Figure S3: This figure relates to Figure 3 and shows by Q-PCR that expression of PFE markers (*PDX1* and *HNF6*) is enriched in PFE (A), while AFE markers (*SOX2* and *TBX1*) is enriched in AFE (B). n=3, error bars = ± S.E.M, **p<0.01.

Figure S4: This figure relates to Figure 6 and shows by qPCR expression of several other cell surface markers and their enrichment in either GFP positive or GFP negative cell populations (A and B). It also shows gene expression analysis of cells at various stages of differentiation revealing that CD56 and CD271 expression peaks in AFE (C) (n=3, error bars = ± S.E.M, # p>0.05, *p<0.05, **p<0.01). It also shows flow cytometry analysis for other candidate cell surface markers that were identified by RNA-seq analysis as being highly expressed in the GFP+ and GFP- populations. Unlike CD56 and CD271 none of these cell surface markers correlated with SOX2-GFP expression (D).

Tables S1-4

Table S1: This table relates to Figure 5 and provides the full data set of the genome wide expression analysis by RNA-seq of 8 day AFE GFP positive and GFP negative cell populations. This table is provided as an Excel spreadsheet.

Table S2: This table relates to Figure 6 and lists all differentially expressed genes that encode cell surface markers. This table is provided as an Excel spreadsheet.

Table S3: This table lists all Taqman primer sets used in this study. These primers were used for Q-PCR analysis shown in Figure 1-5, 7, S3, S4, and S5.

Table S4: This table lists all antibodies used in this study. Antibodies were used for flow cytometry or immuno-fluorescence in Figures 1-5, 7 and S2.

SUPPLEMENTAL EXPERIMENTAL PROCEDURES

Cells and culture conditions. The following media were used: mouse embryonic fibroblast (MEF) and HEK-293T (1X high glucose DMEM, 10% fetal bovine serum, 1% (v/v) L-glutamine penicillin/streptomycin); H9/WA09 hESCs (1X DMEM-F12, 20% (v/v) Knockout Serum Replacement, 1% (v/v) non-essential amino acids, 0.5% (v/v) glutamine, 120 µM 2-mercaptoethanol [Sigma]). All media components are from Life Technologies unless indicated otherwise. H9 hESC lines were maintained on feeder layers of mitotically inactivated MEFs ($2x10^4/cm^2$; Millipore). All hESC cultures were supplemented with 30 ng/ml bFGF (Life Technologies). MEF-CM was produced by culturing MEFs in hESC medium for 24 hr followed by sterile filtering. Cells were routinely passaged with Accutase (Millipore), washed, and replated at a density 4.25 x $10^4/cm^2$.

AAV production and gene targeting. The *SOX2* targeting vector carried a central cassette that included the coding sequence for GFP with a poly-adenylation (pA) signal and a *Neomycin* (*Neo*) resistance gene with a pA under the control of a SV40 promoter. Flanking this central cassette was ~1 kilobase (kb) of genomic DNA from the *SOX2* locus. Primers for the 1kb genomic DNA upstream of the *SOX2* start codon (left homology arm = Probe 1) were: 5'-TGTAAGGTAAGAGAGGAG-3' and 5'-CTCCATCATGTTGTACAT-3'. Primers for the 1kb genomic DNA downstream of the *SOX2* stop codon (right homology arm = Probe 2) were: 5'-TCTCACACATGTGAGG-3' and 5'- TGGATTCTCGGCAGAC-3'. To complete the targeting vector, each construct carried two flanking AAV ITRs. We designed the *SOX2* targeting vector such that the *SOX2* coding region would be replaced with GFP. Supernatants carrying infectious AAV particles were produced as previously described (Hirata et al., 2002). A detailed protocol for AAV production can be found here: vectorcore.salk.edu/protocols/AAV%20Production%20Protocol.doc. Briefly, HEK-293T cells were transfected with plasmids at a 1:1:1 molar ratio of the targeting vector, an adenoviral helper plasmid needed for replication (pXX680), and a plasmid that encodes the cap protein for serotype 2. Previous experiments demonstrated that serotype 2 of AAV (AAV-2) efficiently transduces hESCs (Smith-Arica et al., 2003). Virus particles were harvested from cell extracts by freeze-thawing and trituration. A 15% to 58% iodixanol step gradient was used to purify viral particles by density gradient centrifugation. Quantification of the purified SOX2-GFP AAV-2 virus stocks by Q-PCR indicated concentrations of 9.45 x 10^{11} genome copies (GC) per ml. Prior to infection, H9 hESCs were grown on Matrigel coated plates supplemented with MEF-

conditioned media. H9 cells were infected with the SOX2-GFP AAV-2 supernatants at approximately 10^{10} GC per infection. After 24 hours, virus was removed and G418 (50 µg/mL) was applied for 2 weeks. After 2 weeks, colonies with characteristic hESC morphologies were manually picked and transferred to fresh MEF feeder cells in 96-well plates. Genomic DNA extracted from $G418^R$ clones was analyzed using Southern blot analysis with probes to the left (Probe 1) and right (Probe 2) homology arms. Of the G418 resistant clones analyzed, 26 (72%) were found to carry the GFP-Neo cassette in the *SOX2* locus.

Neural differentiation and neural progenitor cell (NPC) culture. To initiate neural differentiation, H9 hESCs were cultured on Matrigel (BD Biosciences) in MEF-CM supplemented with 30 ng/ml FGF2. Cells were then detached with treatment with Accutase (Millipore) for 5 min and resuspended in neural induction media (1% N2/1% B27/DMEM:F12) supplemented with 5 µM Y-267632 (Stemgent), 50 ng/ml recombinant mouse Noggin (R&D Systems), 0.5 µM Dorsomorphin (Tocris Bioscience). Next, 7.5 x 10^5 cells were pipetted to each well of a 6-well ultra low attachment plates (Corning). The plates were then placed on an orbital shaker set at 95 rpm in a 37°C/5% CO_2 tissue culture incubator. The next day, the cells formed spherical clusters and the media was changed to neural induction media with 50 ng/ml recombinant mouse Noggin and 0.5 µM Dorsomorphin. The media was subsequently changed every other day. After 5 days in suspension culture, EBs were transferred to a 10 cm dish coated (3 x 6 wells per 10 cm dish) with growth factor reduced Matrigel (1:25 in KnockOut DMEM; BD Biosciences) for attachment. The plated EBs were cultured in neural induction media with 50 ng/ml recombinant mouse Noggin and 0.5 µM Dorsomorphin for an additional 7 days. Neural rosettes were dissected under an EVOS (Life Technologies) microscope. Dissected rosettes were incubated in Accutase for 5 min, triturated to single cells with a 1 mL pipet, and plated onto poly-L-ornithine (PLO; 10 µg/mL; Sigma) and mouse laminin (Ln; 5 µg/mL; Sigma) coated dishes at a density of 12,500 cells/cm^2 in neural induction media supplemented with 10 ng/mL mouse FGF2 and 10 ng/ml mouse EGF2 (R&D Systems). For routine maintenance, NPCs were passaged onto PLO/Ln coated plates at a density of 10,000 cells/cm^2 in neural induction media supplemented with 10 ng/mL mouse FGF2 and 10 ng/ml mouse EGF2.

Endoderm differentiation. HESCs were passaged at a density of 2.5 x 10^5 cells/ml onto Matrigel coated plates in order to achieve confluency the following day. HESCs were then gently washed twice with RPMI (Life Technologies) to remove cell debris and residual hESC media.

For definitive endoderm (DE) induction, the medium was changed to RPMI supplemented with 1% (v/v) Gluta-MAX and 100 ng/ml recombinant human Activin A (R&D Systems). Cells were cultured for 3 days, with FBS concentrations at 0% for the first day and 0.2% for the second and third days. Cultures were supplemented with 30 ng/ml purified mouse Wnt3a (Willert et al., 2003) for the first day. For differentiation of anterior foregut endoderm (AFE), DE cells were treated for 5 days with DMEM:F12 media supplemented with 1% B27, 1% N2, 200 ng/ml Noggin, and 10 μM SB-431542 (Tocris). For differentiation to lung progenitor cells (LPCs), AFE cells were treated for 5 days with DMEM:F12 media supplemented with 1% B27, 1% N2, 100 ng/mL Wnt3a (Willert et al., 2003), 10 ng/mL mouse KGF (R&D Systems), 100 ng/mL mouse FGF2, 10 ng/ml mouse BMP4 (R&D Systems), 10 ng/mL mouse FGF10 (R&D Systems), and 10 ng/mL EGF. For differentiation to posterior foregut endoderm (PFE), DE cells were cultured in RPMI with 0.2% FBS supplemented with 50 ng/ml recombinant human KGF for 3 days and then in DMEM with 1× B27, 50 ng/ml recombinant human Noggin, 0.25 μM KAAD-cyclopamine (Tocris), and 2 μM retinoic acid (Sigma) for 3 days.

Quantitative reverse transcription PCR (Q-PCR). RNA was isolated from cells using TRIzol (Life Technologies), and treated with DNase I (Life Technologies) to remove traces of genomic DNA. Reverse transcription was performed by means of qScript cDNA Supermix (Quanta Biosciences). Q-PCR was carried out using TaqMan probes (Life Technologies) and TaqMan Fast Universal PCR Master Mix (Life Technologies) on a 7900HT Real Time PCR machine (Life Technologies), with a 10 min gradient to 95°C followed by 40 cycles at 95°C for 15s and 60°C for 1 min. Taqman gene expression assay primers (Life Technologies; **Table S3**) were used. Gene expression was normalized to 18S rRNA levels. Delta Ct values were calculated as $C_t^{target} – C_t^{18s}$. All experiments were performed with three technical replicates. Relative fold changes in gene expression were calculated using the $2^{-\Delta\Delta Ct}$ method. Data are presented as the average of the biological replicates ± standard error of the mean.

Immunofluorescence (IF). Cultures were gently washed twice with staining buffer (PBS w/ 1% (w/v) BSA) prior to fixation. Cultures were then fixed for 15 min at room temperature (RT) with fresh paraformaldeyde (4% (w/v)). The cultures were washed twice with staining buffer and permeabilized with 0.2% (v/v) Triton-X-100 in stain buffer for 20 min at 4°C. Cultures were then washed twice with staining buffer. Primary antibodies were incubated overnight at 4°C and then washed twice with stain buffer at RT. Secondary antibodies were incubated at RT for 1 hr. Antibodies are listed in **Table S4**. Nucleic acids were stained for DNA with Hoechst 33342 (2

μg/ml; Life Technologies) for 5 min at room temperature. Imaging of fixed and stained cells was performed using an automated confocal microscope (Olympus Fluoview 1000 with motorized stage and incubation chamber). Quantification of images was performed by counting a minimum of 9 fields at 20x magnification.

Flow cytometry and cell replating. Cells were dissociated with Accutase for 5 min at 37°C, triturated, and passed through a 40 μm cell strainer. Cells were washed twice with FACS buffer (PBS, 10 mM EDTA, and 2% FBS) and resuspended at a maximum concentration of 5×10^6 cells per 100 μl. One test volume of antibody was added for each 100 μl cell suspension (**Figure 4**). Cells were stained for 30 min on ice, washed, and resuspended in stain buffer. Cells were analyzed and sorted with a FACSCanto or FACSAria2 (BD Biosciences). FACS data was analyzed with FACSDiva software (BD Biosciences). For replating experiments, cells were stained with appropriate antibodies and sorted into FACS buffer with 10 nM Y27632 (Stemgent). Sorted cells were replated at the appropriate density and media with 10 nM Y27632. Isotype negative controls are listed in **Table S4**. For sorting experiments in which cells were separated on the basis of GFP expression, wild-type (WT) non-fluorescing cells were used as a negative control.

High throughput RNA sequencing (RNA-seq). Total RNA from FACS sorted (FACS Aria 2) SOX2-GFP$^+$ and SOX2-GFP$^-$ AFE cells were isolated, depleted of genomic DNA and rRNA and fragmented to ~200 bp by RNase III. After ligating the Adaptor Mix, fragmented RNA was converted to the first strand cDNA by ArrayScript Reverse Transcriptase (Ambion), size selected (100-200bp) by gel electrophoresis, and amplified by PCR using adaptor-specific primers. Deep sequencing was performed on an Illumina Genome Analyzer II (Next Genartion Sequencing Core Facility at the Scripps Research Institute). Differential gene expression analysis of RNA-seq experiments was performed with TopHat and Cufflinks as previously described (Trapnell et al., 2010; Trapnell et al., 2012). Briefly, raw reads for the sorted SOX2-GFP$^+$ and SOX2-GFP$^-$ AFE samples were filtered, mapped, and aligned to the reference human genome (hg19) with TopHat. Cuffmerge was used to merge a total of 87,978,934 reads and 84,102,366 reads that were obtained from two biologically independent SOX2-GFP$^+$ and SOX2-GFP$^-$ samples, respectively. Finally, Cuffdiff was used to calculate gene expression levels and identify statistically significant differences in gene expression. Reads per kilobase of exon per million mapped reads (RPKM) were calculated for each gene and used as an estimate of expression levels.

LEGENDS FOR SUPPLEMENTAL FIGURES

Supplemental Figure S1, related to Figure 1. Characterization of SOX2-GFP clones. A. Southern blot analysis of EcoRI digested genomic DNA with Probes 1 and 2 (see Figure 1A). A red star indicates a clone that showed a band expected for a targeted insertion. B. Uncropped image of Southern blot shown in Figure 1B. C. Table summarizing sizes of bands expected for each Southern blot.

Supplemental Figure S2, related to Figure 1. Characterization of clone hSOX2-23. A. Flow cytometry analysis of GFP expression in hSOX2-23 (positive clone) and hSOX2-25 (negative clone) hESCs. B. Quantitative reverse transcription PCR (Q-PCR) of H9 (WT H9) and two G418 selected clones (Clone 23 and Clone 25). Despite loss of one copy of the *SOX2* gene in Clone 23, expression of the pluripotency genes *SOX2*, *OCT4* and *NANOG* is unaffected. N.S. = not significant (n=3 independent experiments). C. Immunofluorescence analysis of GFP and SOX2 showed colocalization in undifferentiated hESCs. D. Phase contrast image of undifferentiated hSOX2-23 hESCs demonstrates that cells exhibit a morphology characteristic of undifferentiated hESCs. E. hSOX2-23 hESCs express the pluripotency marker NANOG. F. GFP+ and GFP- populations were isolated using fluorescence-based cell sorting and replated. The GFP+ population retains the characteristic morphology of pluripotent cells while the GFP- population acquired a fibroblast-like morphology.

Supplemental Figure S3, related to Figure 3. Gene expression analysis of hESC derived anterior and posterior foregut endoderm. A. PFE markers *HNF6* and *PDX1* are not expressed in hESCs differentiated to anterior foregut endoderm (AFE) (n=3 independent experiments, mean ± S.E.M). B. Conversely, markers *SOX2* and *TBX1* were enriched in hESCs differentiated to AFE but not posterior foregut endoderm (PFE) (n=3 independent experiments, mean ± S.E.M).

Supplemental Figure S4, related to Figure 6. Validation of cell surface markers differentially expressed in GFP^+ and GFP^- AFE cells. A. Q-PCR analysis of candidate cell surface markers that were identified to be differentially expressed in GFP^+ and GFP^- cells by genome-wide expression analysis (n=3 independent experiments, mean ± S.E.M). B. Q-PCR analysis of *CD56* and *CD271* reveals a slight enrichment of these genes in the GFP^+ versus

GFP$^-$ populations (n=3 independent experiments, mean ± S.E.M). C. Flow cytometry analysis for additional candidate cell surface markers identified by RNA-seq analysis as being highly expressed in the GFP$^+$ and GFP$^-$ populations. In contrast to CD56 and CD271, none of these cell surface markers correlated with SOX2-GFP expression. D. Q-PCR analysis of CD56 and CD271 at various stages of differentiation reveals that CD56 and CD271 expression peaks in AFE. (n=3 independent experiments, error bars = ± S.E.M, # p>0.05, *p<0.05, **p<0.01.

Supplemental Table S1, related to Figure 5. RNA-seq data of GFP$^+$ and GFP$^-$ day 8 AFE cells. Provided separately in an Excel spreadsheet "SCR-D-13-00079R1_SupplTable1".

Supplemental Table S2, related to Figure 6. RNA-seq data of cell surface markers differentially expressed in GFP$^+$ and GFP$^-$ day 8 AFE cells. Provided separately in an Excel spreadsheet "SCR-D-13-00079R1_SupplTable2".

Supplemental Table S3. TaqMan gene expression assays used in this study.

Supplemental Table S4. Antibodies used in this study.

Supplemental Figure S1

A

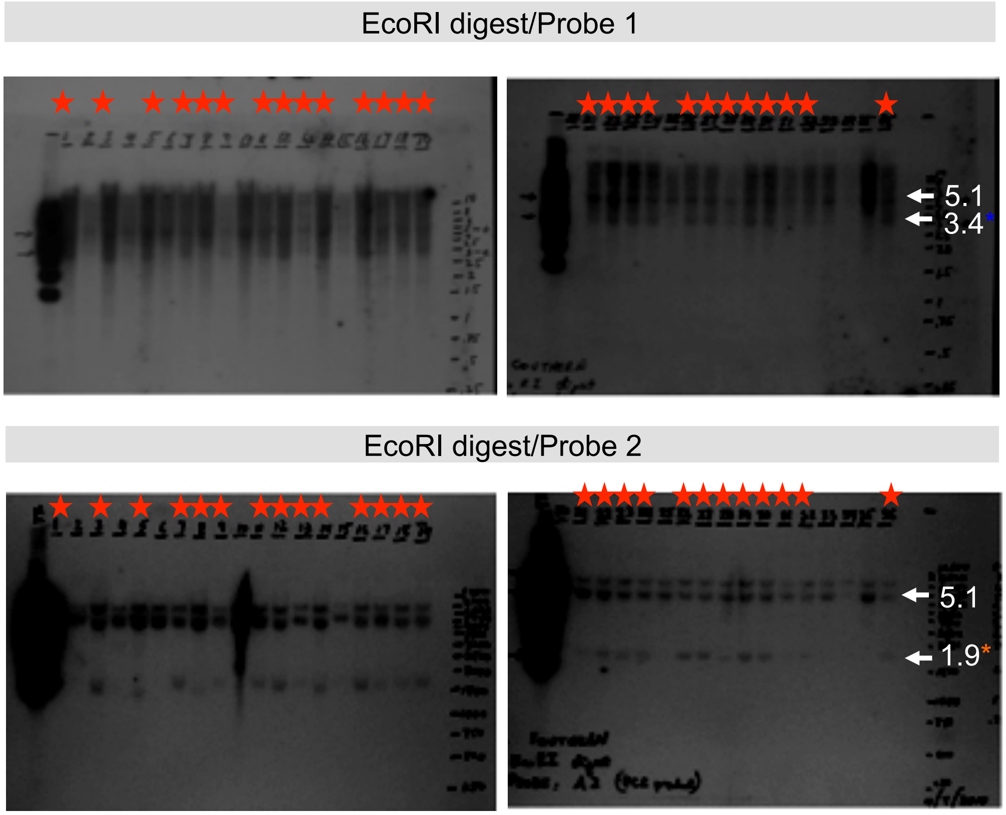

26 positive clones★/36 total G418^R clones = 72% targeting efficiency

B

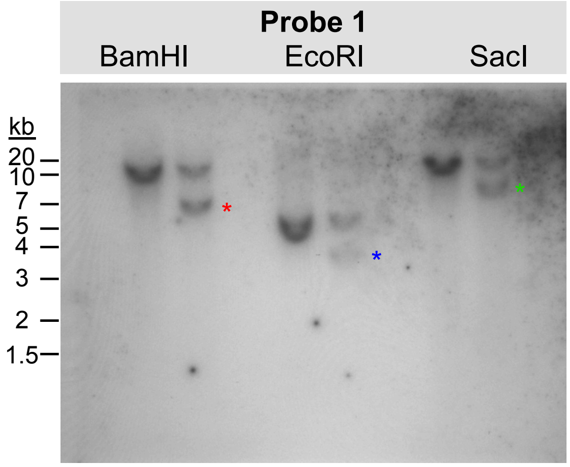

C

	Probe 1	Probe 2
**BamHI**		
Wildtype	12.8	Not shown
Targeted	6.3*	Not shown
**EcoRI**		
Wildtype	5.1	5.1
Targeted	3.4*	1.9*
**SacI**		
Wildtype	13.3	Not shown
Targeted	7.7*	Not shown

Supplemental Figure S2

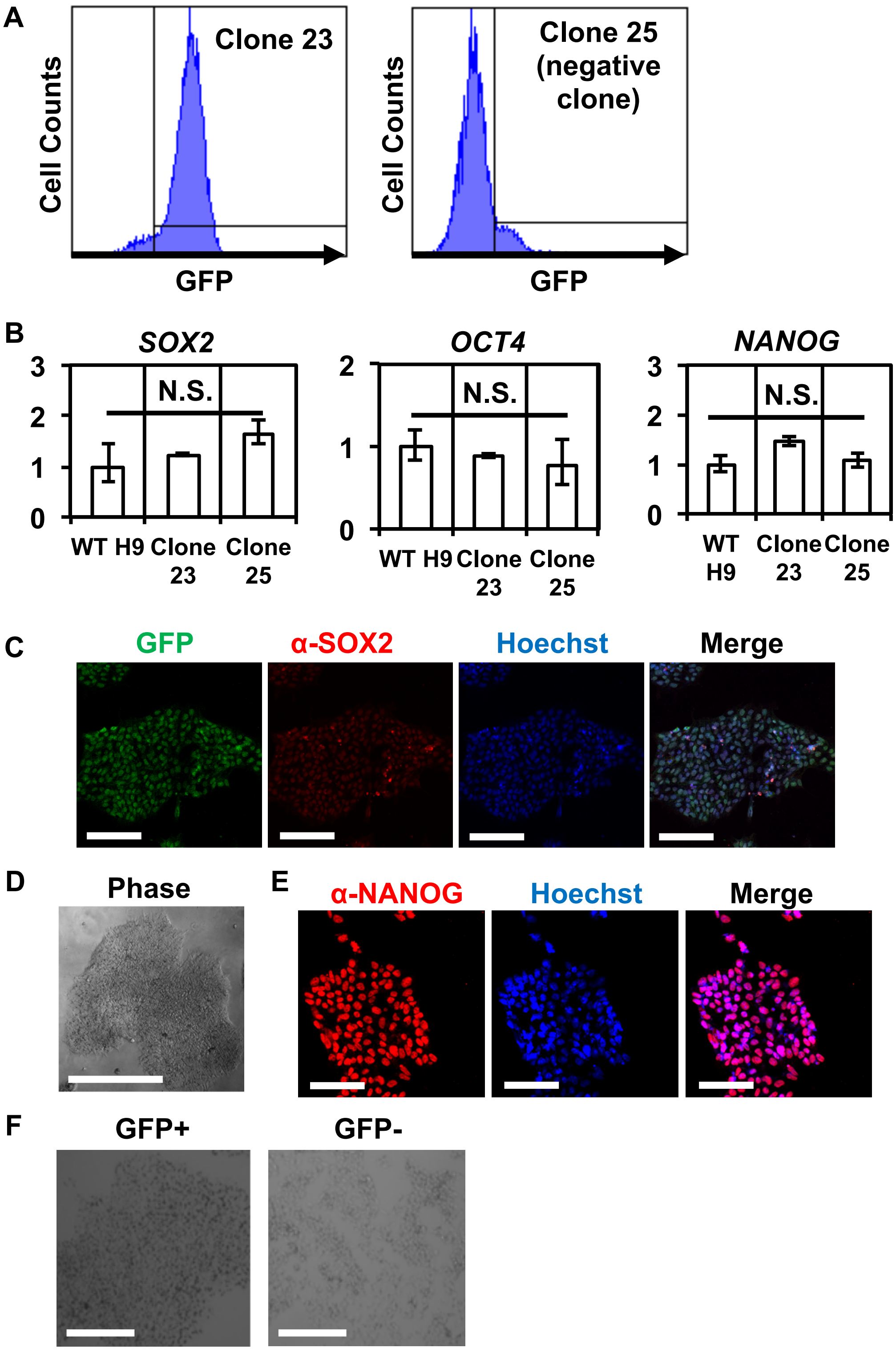

Supplemental Figure S3

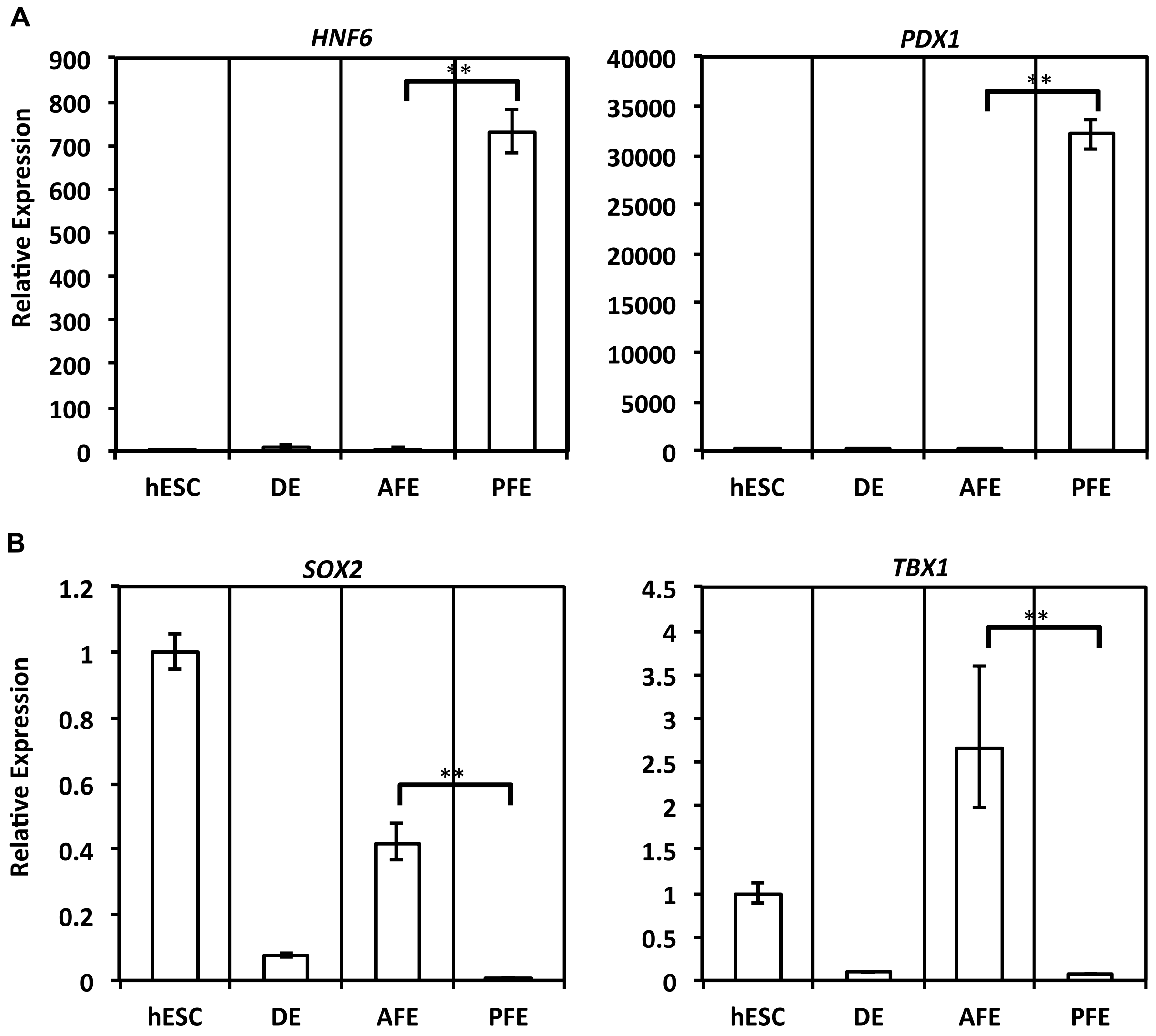

Supplemental Figure S4

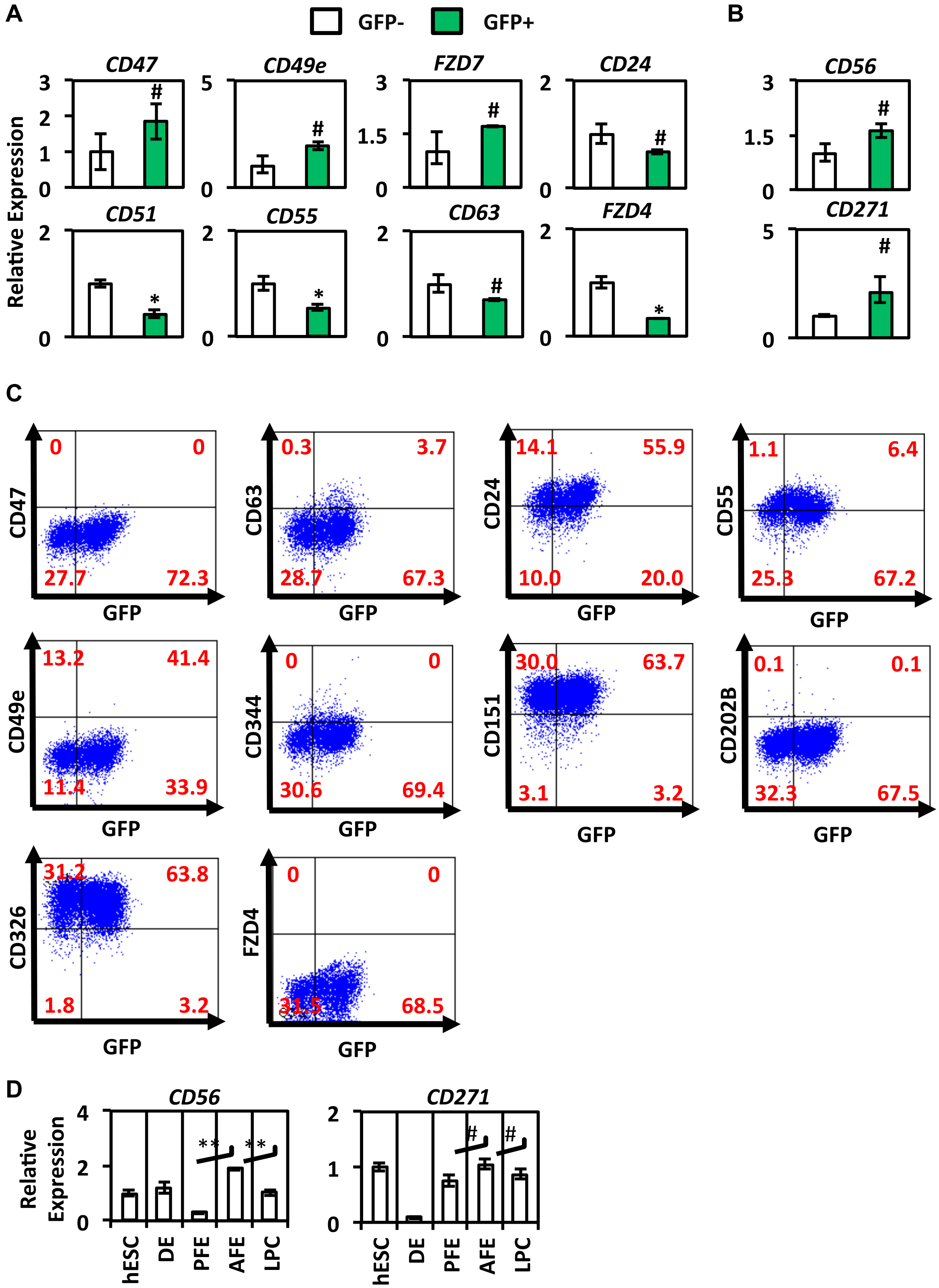

Supplemental Tables 1 and 2:

See Excel spreadsheet “SCR-D-13-00079R1_SupplTables-1” and “SCR-D-13-00079R1_SupplTables-2”

Supplemental Table 3

Gene	ABI Assay
18s	Hs99999901_s1
SOX2	Hs01053049_s1
OCT4	Hs00742896_s1
NANOG	Hs02387400_g1
PAX6	HS01088112_m1
SOX1	HS01057642_s1
NESTIN	Hs00707120_s1
SOX17	Hs00751752_s1
FOXA2	Hs0023764_m1
TBX1	Hs00271949_m1
NKX2.1	Hs00968940_m1
SOX9	Hs01001342_g1
PAX9	Hs00196354_m1
HOXA1	Hs00939046_m1
HOXA2	Hs00534579_m1
HNF1B	Hs01001602_m1
HNF4A	Hs00230853_m1
GATA6	Hs00232018_m1
CDX2	Hs01078080_m1
PDX1	Hs00236830_m1
CD56	Hs00941830_m1
CD271	Hs00609977_m1
HNF6	Hs00413554_m1
CD47	Hs00179953_m1
CD49e	Hs01547673_m1
FZD7	Hs00275833_s1
CD24	Hs00175568_m1
CD51	Hs00233808_m1
CD55	Hs00892618_m1
CD63	Hs00156390_m1
FZD4	Hs00201853_m1

Supplemental Table 4

Antibody	Vendor	Catalog #	Concentration Used
Goat anti-SOX2	Santa Cruz	sc-17320	1:50
Rabbit anti-NANOG	Santa Cruz	sc-33759	1:50
Rabbit anti-OCT4	Santa Cruz	sc-9081	1:50
Rabbit-anti NKX2.1	Abcam	ab76013	1:200
Alexa 647 Donkey anti-goat	Life Technologies	A-21447	1:200
Alexa 647 Donkey anti-rabbit	Life Technologies	A-21244	1:200
PE anti-SOX2	BD	560291	20 ul per test
PerCp-Cy5.5 anti-SOX1	BD	561549	5 ul per test
Alexa 647 anti-TRA-1_81	BD	560793	20 ul per test
PE anti-CD49e	BD	555617	20 ul per test
PE anti-CD47	BD	556046	20 ul per test
PE anti-CD55	BD	561901	20 ul per test
PE anti-CD63	BD	557305	20 ul per test
PE anti-CD24	BD	560991	20 ul per test
PE anti-CD56	BioLegend	355503	5 ul per test
PE anti-CD344	BioLegend	124009	5 ul per test
PE anti-CD334	BioLegend	324207	5 ul per test
PE anti-CD151	BioLegend	326605	5 ul per test
APC anti-CD202	BioLegend	324305	5 ul per test
APC anti-CD326	BioLegend	350405	5 ul per test
APC anti-CD271	BioLegend	345107	5 ul per test
PE IgG1 Isotype Control	BioLegend	400113	5 ul per test
PE IgG2a Isotype Control	BD	555574	20 ul per test
APC IgG1 Isotype Control	BioLegend	400121	5 ul per test
Alexa 647 IgM Isotype Control	BD	560806	20 ul per test
PercCp-Cy5.5 IgG1 Isotype Control	BD	550794	5 ul per test
